# Buffalo Medical College

**Published:** 1879-11

**Authors:** 


### THE BUFFALO MEDICAL COLLEGE.

THE regular session of 1879–80 opened Oct. 8th, Prof. J. F. Miner delivering the introductory address. The attendance of the profession, and also of students, was large. The address was one of the ablest efforts of our late editorial confrère. The medical class is an unusually large and promising one. Its numerical proportions have peculiar significance in view of the movement to extend the course, and to establish a system of more frequent and thorough examinations. This improved feature, we took occasion to refer to in commendatory terms in our last number. It is in response to the demand of the profession for a higher standard of medical education, and we heartily indorse the efforts of the Buffalo Medical College in this as in every effort to this end. We trust, also, that its faculty, who are justly ranked among our most liberal and progressive men, will stop only when they have established a complete graded course of medical study, such as Harvard now offers, and Bellevue intends to establish.

